# Gut Microbiota in Women with Eating Disorders: A New Frontier in Pathophysiology and Treatment

**DOI:** 10.3390/nu17142316

**Published:** 2025-07-14

**Authors:** Giuseppe Marano, Sara Rossi, Greta Sfratta, Mariateresa Acanfora, Maria Benedetta Anesini, Gianandrea Traversi, Francesco Maria Lisci, Lucio Rinaldi, Roberto Pola, Antonio Gasbarrini, Gabriele Sani, Eleonora Gaetani, Marianna Mazza

**Affiliations:** 1 Unit of Psychiatry, Fondazione Policlinico Universitario Agostino Gemelli IRCCS, 00168 Rome, Italy; 2 Department of Neurosciences, Università Cattolica del Sacro Cuore, 00168 Rome, Italy; 3 Unit of Medical Genetics, Department of Laboratory Medicine, Ospedale Isola Tiberina-Gemelli Isola, 00186 Rome, Italy; gianandrea.traversi@gmail.com; 4 Section of Internal Medicine and Thromboembolic Diseases, Department of Internal Medicine, Fondazione Policlinico Universitario Agostino Gemelli IRCCS, Università Cattolica del Sacro Cuore, 00168 Rome, Italy; 5 Internal Medicine and Gastroenterology, Fondazione Policlinico Universitario Agostino Gemelli IRCCS, 00168 Rome, Italy; 6 Department of Translational Medicine and Surgery, Fondazione Policlinico Universitario Agostino Gemelli IRCCS, Università Cattolica del Sacro Cuore, 00168 Rome, Italy; eleonora.gaetani@unicatt.it (E.G.); mariannamazza@hotmail.com (M.M.); 7 Unit of Internal Medicine, Cristo Re Hospital, 00167 Rome, Italy

**Keywords:** eating disorders, anorexia nervosa, bulimia nervosa, binge eating disorder, gut microbiota, pathophysiology, microbiota-targeted interventions, women

## Abstract

Emerging evidence highlights the critical role of the gut microbiota in the development and progression of eating disorders (EDs), particularly in women, who are more frequently affected by these conditions. Women with anorexia nervosa, bulimia nervosa, and binge eating disorder exhibit distinct alterations in gut microbiota composition compared to healthy controls. These alterations, collectively termed dysbiosis, involve reduced microbial diversity and shifts in key bacterial populations responsible for regulating metabolism, inflammation, and gut–brain signaling. The gut microbiota is known to influence appetite regulation, mood, and stress responses—factors closely implicated in the pathogenesis of EDs. In women, hormonal fluctuations related to menstruation, pregnancy, and menopause may further modulate gut microbial profiles, potentially compounding vulnerabilities to disordered eating. Moreover, the restrictive eating patterns, purging behaviors, and altered dietary intake often observed in women with EDs exacerbate microbial imbalances, contributing to intestinal permeability, low-grade inflammation, and disturbances in neurotransmitter production. This evolving understanding suggests that microbiota-targeted therapies, such as probiotics, prebiotics, dietary modulation, and fecal microbiota transplantation (FMT), could complement conventional psychological and pharmacological treatments in women with EDs. Furthermore, precision nutrition and personalized microbiome-based interventions tailored to an individual’s microbial and metabolic profile offer promising avenues for improving treatment efficacy, even though these approaches remain exploratory and their clinical applicability has yet to be fully validated. Future research should focus on sex-specific microbial signatures, causal mechanisms, and microbiota-based interventions to enhance personalized treatment for women struggling with eating disorders.

## 1. Introduction

According to the DSM-5-TR, feeding and eating disorders include a group of disorders in which the alteration of eating and eating-related behavior is the crucial element, leading to disturbances in psychological well-being and physical health. Anorexia nervosa (AN), bulimia nervosa (BN), and binge eating disorder (BED) are the most commonly known disorders, having higher prevalence in women compared to men [1]. Despite significant advancements in psychotherapeutic and pharmacological interventions in recent years, treatment outcomes remain modest, with high relapse rates and persistent challenges in long-term recovery [2]. Research is shedding light on the gut–brain axis as a pivotal player in the pathophysiology of EDs.

Gut microbiota plays a fundamental role in regulating metabolism, immune responses, neurotransmitter synthesis, and neurobiological mechanisms [3]. In women with EDs, emerging evidence indicates that the microbial community is markedly altered, posing the basis for different eating behavior phenotypes [4]. It can be further influenced by female-specific hormonal cycles, stress, and even sociocultural pressures concerning body image and dietary norms [5].

The resulting disturbances in gut–brain signaling, intestinal permeability, and inflammation may not only exacerbate the core features of EDs but also perpetuate comorbid mood and anxiety disorders, creating a situation in which eating disorders and gut microbiota contribute to worsen each other [6].

This narrative review uniquely addresses the intersection between gut microbiota and eating disorders in women, with a specific focus on sex-specific microbial signatures and hormonal influences across the female lifespan, including puberty, pregnancy, and menopause. While several prior reviews have explored gut–brain interactions or microbiota alterations in EDs, none have comprehensively examined sex-based differences in microbiome composition, immune responses, or hormonal modulation, nor have they synthesized emerging evidence on microbiota-targeted interventions with a female-centered perspective. By bridging findings from human and animal studies, this work advances a personalized, microbiota-informed model of ED pathophysiology and treatment, offering a novel translational framework for future clinical applications.

## 2. Gut Microbiota and Mental Health: Functional Roles, Gut–Brain Axis Interactions, and Sex-Related Differences Across the Lifespan

### 2.1. Gut Microbiota and Mental Health

The microbial composition in the gut plays a critical role in emotional and cognitive well-being; indeed, the connection between gut microbiota and mental health has captured the attention of researchers [7]. When there is an imbalance in these microbial communities, this is a condition known as dysbiosis; it can be linked to various mental health issues, for example, depression and anxiety [8]. The speed at which food moves through our digestive system, defined as the gut transit time, can affect the diversity and structure of these microbes. Diets high in fat and sugar tend to lead to dysbiosis, while a diet rich in fiber, prebiotics, and probiotics helps create a healthier microbial environment [9]. So, our diet, stress levels, and medications can change our gut microbiota and our mental health. Ongoing research is exploring how diet and probiotics might help manage mental health conditions, stressing the importance of having a stable and diverse gut microbiome for maintaining psychological well-being [10].

### 2.2. Gut Microbiota Composition

The gut microbiota is a complex and diverse ecosystem of microorganisms, including bacteria, archaea, viruses, and eukaryotic microbes, residing in the gastrointestinal tract [11]. Microbial cells are roughly equal in number to human cells (∼3.8 × 10^13^ vs. 3 × 10^13^), yet their genetic content exceeds that of human genes by 100–200 times [12].

Among the more than 1,500 identified species, Firmicutes and Bacteroidetes dominate, comprising over 90% of the gut microbiota, followed by Proteobacteria, Actinobacteria, Verrucomicrobia, and Fusobacteria [11,13]. Firmicutes includes over 200 genera, such as *Clostridium*, *Lactobacillus*, *Bacillus*, and *Enterococcus*, while Bacteroidetes mainly includes *Bacteroides* and *Prevotella* [13,14].

This community adapts to local conditions (e.g., pH, nutrient availability) and contains both beneficial and potentially harmful species. Symbiotic genera like *Bifidobacterium* and *Lactobacillus* are widely recognized for their health-promoting roles and are commonly used in probiotics [15]. Advancements in sequencing technologies, especially 16S rRNA gene analysis, have improved our ability to study gut microbes and develop personalized therapeutic approaches [16].

Beyond digestion and immune regulation, the microbiota also influences mental health via the gut–brain axis, a bidirectional communication system between the gut and the brain [17]. Its composition varies widely between individuals, influenced by factors such as diet, genetics, age, lifestyle, antibiotic use, and mode of birth [14].

### 2.3. Microbial Distribution Along the Gastrointestinal Tract

The gastrointestinal tract, which stretches from the mouth all the way to the anus, is made up of several distinct segments, each with its own anatomy and function. These include the oral cavity, pharynx, esophagus, stomach, small intestine (which consists of the duodenum, jejunum, and ileum), large intestine (including the cecum, colon, and rectum), and the anal canal [18]. Each of these areas creates a unique microbial environment shaped by factors like local pH, oxygen levels, secretions, and the types of nutrients available [13].

In the stomach, the acidic conditions keep most microbes at bay, leading to a relatively low bacterial density of about ~10^3^ CFU/mL. However, Firmicutes and Proteobacteria still manage to thrive here, particularly genera such as *Streptococcus* and *Prevotella* [14]. It is worth noting that an infection with *Helicobacter pylori* can significantly alter the gastric microbiota, which can lead to issues like gastritis, peptic ulcers, and even gastric cancer [19].

As we move into the duodenum, the quick passage of food and low oxygen levels result in a decrease in both the number and variety of microbes, with Firmicutes and Actinobacteria taking the lead [14]. The jejunum sees a rise in microbial diversity, where facultative anaerobes like Lactobacilli, Enterococci, and Streptococci become more prevalent.

The most complex and varied microbial community, predominantly consisting of Firmicutes and Bacteroidetes, exists in the colon and thrives under anaerobic conditions [14]. In this location, an increase in transit time elevates fecal pH and reduces water content, causing a shift in the metabolism of bacteria from fermenting carbohydrates to fermenting proteins. This shift can increase the harmful byproducts of branched-chain fatty acids and hydrogen sulfide while decreasing beneficial byproducts like SCFAs produced from carbohydrate fermentation [20].

### 2.4. The Role of the Microbiota in Regulating the Gut–Brain Axis

The gut microbiota contributes to host cellular homeostasis and helps regulate the gut–brain axis, fulfilling numerous functions [21,22,23].

The microbiota assists in digestion, including the absorption of nutrients in the intestines. It also aids in maintaining the intestinal barrier by controlling cellular metabolism, immune response development, and the enhancement of immune defenses [24]. On the other hand, if intestinal permeability regulation goes wrong, innate immune system activation can occur, leading to systemic neuroinflammation. This inflammatory condition can increase the likelihood of experiencing stress as well as psychiatric problems. Inflammatory exudation present in the gut epithelium is efficiently controlled by the colon-residing bacteria, which activate anti-inflammatory cascades, secrete protective antimicrobial peptides and mucus, and participate in the repair of gut epithelial tissues [25].

Among the variables that can influence the composition and function of our microbiota are age, place of residence, diet, drugs, exposure to toxins, infectious agents, and even our genes [26]. Moreover, newborns’ microbiota is shaped by the type of delivery at birth and nursing [27].

Recent studies have shown that our gut microbiota is quite important in generating several neurotransmitters. For instance, *Bifidobacterium* and *Lactobacillus* are known to generate γ-aminobutyric acid (GABA), which boosts inhibitory signaling in the brain. Similarly, *Lactobacillus* and *Oscillibacter* enhance the expression of tryptophan synthase, leading to increased serotonin production. Among other things, vagus nerve stimulation and immune system activity affect the production of these neurotransmitters. Activation of afferent vagal routes via G-protein-coupled receptors or histone deacetylases can influence the behavior of immune cells, including monocytes, macrophages, neutrophils, dendritic cells, and T cells, in terms of their function and recruiting ability [28,29].

Thanks to its extensive network of nerve endings discovered in the intestinal mucosa and submucosa—which make up roughly 90% of its overall nerve endings—the vagus nerve is absolutely important for regulating the gut–brain axis. Beyond its gut activities, the vagus nerve also influences memory, emotional control, and cognitive processes via its contacts with the cerebral cortex, amygdala, and hippocampus [30]. It oversees gastrointestinal activities, including the decreasing of gastric emptying and the stimulation of digestive enzyme release, which are critical for digestion and nutrient absorption. It also detects microbial metabolites through several pathways, including afferent sensory mechanisms and receptor systems on its surface like serotonin (5-HT) and dopamine receptors, Toll-like receptor 4 (TLR4), and free fatty acid receptors. Disturbances in this signaling network, as shown in neuroimaging studies, can result in functional changes associated with mental health conditions like substance use disorders [31], mood disorders, and eating disorders [31,32].

From the early years, the intestinal microbiota is vital in sculpting immune responses on several levels. It encourages innate immunity by activating gut-associated lymphoid tissue, while interactions between bacterial components and receptors such as TLR9 and inflammasomes on epithelial and immune cells trigger both localized and systemic immune responses [33]. When the immune system fails to distinguish self from non-self antigens, a pathological process called autoimmunity occurs, involving an adaptive immune response against self-antigens. This breakdown of self-tolerance affects both innate and adaptive immunity, disrupting intercellular signaling and contributing to autoimmune diseases [24,34].

T and B lymphocytes, which coordinate immune responses by displaying molecular signals called Pathogen-Associated Molecular Patterns (PAMPs), lie at the basis of identifying pathogens. Whether through necrosis or apoptosis, cell death releases endogenous molecules called Damage-Associated Molecular Patterns (DAMPs) [24,34]. Pattern recognition receptors (PRRs) gather these and start inflammatory cascades. The gut is where similar processes happen; intestinal epithelial cells use Toll-like receptors, Nod-like receptors, and helicases in combination to start immunological reactions. When the immune system is incorrectly triggered by stimuli that are typically benign, autoimmune responses can result [35].

Recent clinical observations and research are increasingly uncovering a significant connection between autoimmune processes and psychiatric disorders [36]. This relationship may involve changes in how brain proteins are presented to the immune system, molecular mimicry, and the production of autoantibodies that mistakenly target neuronal structures. These mechanisms are thought to play a role in neurological conditions like Parkinson’s disease and multiple sclerosis [37,38].

Many variables affect the emergence of autoimmunity. Although genetic predisposition is quite important in identifying who is vulnerable, environmental variables, including stressors, exposure to toxic compounds, gut microbiome imbalances, and pathogen infections, greatly influence autoimmune reactions [34].

The metabolism of particular metabolites and neurotransmitter precursors, many of which come from gut bacteria, can be thrown off balance when the immune system is not working properly. For instance, in autoimmune diseases, the metabolism of tryptophan, a precursor to serotonin, is compromised, resulting in greater amounts of kynurenine, a substance also created by intestinal bacteria. Enhanced kynurenine metabolism leads to the creation of kynurenic acid and quinolinic acid, both of which can affect dopamine and GABA secretion as well as impact synaptic plasticity and general brain function [39]. Depending on their concentration, these metabolites have impacts: at normal levels, kynurenic acid protects neurons by obstructing NMDA receptors; high levels of quinolinic acid are linked with cognitive impairment caused by synaptic dysfunction [40]. Some studies even suggest that the excitotoxicity of quinolinic acid may further impede synaptic plasticity [41].

### 2.5. Sex Differences in the Intestinal Microbiota Throughout Life

The composition of the human intestinal microbiota is not uniform but rather presents significant sex-related variations [42]. These differences influence various aspects of health, from immunity to the metabolization of sex hormones, and manifest themselves distinctly during different stages of life.

#### 2.5.1. Sex-Related Differences in Gut Microbiota Composition During Childhood (1–12 Years)

During childhood, the introduction of solid foods leads to a microbiota composition that increasingly resembles that of adults, although it remains less stable compared to adult subjects. This period is relevant for examining sex differences, as hormones distinctly contribute to the physical development [43,44,45,46].

Researchers discovered in a study of 277 Colombian children between the ages of 1 and 5 that their microbiota is molded by age, health, where they live, and sex. For example, in healthy male children, *Bifidobacterium* had a strong favorable link with *Lactobacillus*, a relationship not observed in children experiencing diarrhea [47].

- Sex-dependent effects of vitamin A supplementation

Maintaining the health of our intestines and enhancing our mucosal immunity depend in part on vitamin A (VA) [48]. A study involving 306 infants in Bangladesh revealed that males had lower levels of *Bifidobacterium* compared to their female counterparts during the early months of life (6–15 weeks). However, after receiving VA supplementation, the male children showed a notable increase in *Bifidobacterium*, a change that was not seen in the females [49]. This difference might be linked to how testosterone interacts with vitamin A-related signaling pathways [50] and the early rise of DHEA levels in males [51]. Plus, *Bifidobacterium* could help boost immune responses by increasing the number of CD103(+) dendritic cells in the lamina propria [52].

- Temperamental traits and infant microbiota

Behavioral characteristics such as temperament, which are influenced by parental care, have been linked to microbiota composition [53]. In a study of 77 children aged 18 to 27 months, researchers found links between temperament traits—like surgency and fear reactivity—and microbiota composition, although they did not find any differences based on sex [54]. Similarly, a study by Hollister et al. [55] involving children aged 7 to 12 years also reported no sex differences in microbiota structure, though they did observe some minor effects related to ethnicity.

#### 2.5.2. Sex-Related Differences in Gut Microbiota Composition During Adolescence (12–17 Years)

Adolescence is a crucial period defined by major hormonal, behavioral, and neurobiological changes [56], all of which might impact the gut microbiota. A study of adolescent twins, 13 to 17 years old, showed that opposite-sex twins had more variation in their microbial makeup than same-sex twins, which suggests the influence of sex hormones [26]. Another study [57] reported no significant differences in alpha and beta diversity between prepubertal and pubertal individuals but observed some taxonomic shifts with the progression of puberty, notably, a decrease in *Clostridiales* and an increase in *Betaproteobacteria*. Additionally, testosterone levels were found to correlate with the relative abundance of specific taxa such as *Adlercreutzia*, *Ruminococcus*, and *Clostridium*.

Animal studies provide complementary insights, showing that sex-related differences in microbiota tend to emerge during puberty and may diminish following gonadectomy. For instance, in post-pubertal NOD mice, females exhibited greater alpha diversity compared to males, who were enriched in *Porphyromonadaceae*, *Lactobacillaceae*, and *Enterobacteriaceae* [58]. Moreover, the transfer of male microbiota into young female mice was associated with an increase in the recipients’ testosterone levels [59]. While these findings suggest possible bidirectional interactions between sex hormones and gut microbiota, further studies are needed to clarify whether such changes are causally driven by hormonal fluctuations or reflect more complex, developmentally regulated processes.

#### 2.5.3. Sex-Related Differences in Gut Microbiota Composition During Adulthood

Although the gut microbiota tends to stabilize in adulthood, it remains susceptible to various modulatory factors such as diet, antibiotics, disease, and aging [60]. Several studies report consistent sex-related differences in microbial composition. In a small cohort, Dominianni et al. [61] found associations between sex and specific microbial profiles. Mueller et al. observed higher levels of the Bacteroides–*Prevotella* group in adult males, while older women generally presented with greater microbial diversity, including higher levels of *Akkermansia* and *Ruminococcaceae* [62]. In a Japanese cohort, men showed a higher abundance of *Prevotellaceae*, *Megamonas*, and *Megasphaera*, whereas *Ruminococcus*, *Bifidobacterium*, and *Akkermansia* were more prevalent in women.

The composition of *Clostridium* species appears to vary considerably both in newborns and in adult females, potentially reflecting estrogen fluctuations over the lifespan [63]. A large multicenter study (USA, UK, Colombia, China) showed that women aged 20–45 years had higher alpha diversity compared to men, a distinction that diminished after age 60, possibly in connection with menopause and the associated decline in estrogen levels [64,65]. In another Japanese population study of 516 adults, *Prevotella* and *Fusobacterium* were more abundant in males, whereas *Bifidobacterium* and *Akkermansia* predominated in females [66]. Although sex-stratified data are lacking in some populations, interesting patterns have emerged: for instance, centenarians exhibited greater levels of *Roseburia* and *Escherichia* compared to older non-centenarians, though no significant sex-based differences were reported [67]. Lastly, *Holdemanella* and *Gemmiger* show negative correlations with body fat distribution across sexes [68]. Notably, *Holdemanella* produces anti-inflammatory fatty acids [69], which may counter androgen-dependent inflammatory responses in males [70]. However, further studies are necessary to determine whether microbial profiles directly mediate sex-specific metabolic or immunological outcomes.

## 3. Microbiota Alterations in Eating Disorders

### 3.1. Anorexia Nervosa: Microbiota Alterations in Undernutrition

AN is an eating disorder characterized by a restriction of energy intake relative to physiological requirements, leading to significantly low body weight. Patients typically present with an intense fear of weight gain and a distorted body image, often demonstrating an impaired ability to recognize the seriousness of their underweight status [71]. Lifetime prevalence estimates indicate that AN affects up to 4% of females and 0.3% of males, reflecting a pronounced gender disparity [72]. The disorder primarily emerges during adolescence, peaking around age 15; however, a downward trend in the age of onset has been observed, with rare cases reported as early as 8 years old [73]. AN is associated with a mortality rate of 5.1 deaths per 1000 person-years, nearly six times higher than that of age-matched individuals without AN. Notably, approximately 25% of deaths among individuals with AN are attributed to suicide [74].

Gut microbiota disturbances have recently been proposed as contributing factors in the complex pathogenesis of EDs, as shown in Table 1.

Infections with viral and bacterial pathogens, particularly group A *β-haemolytic streptococcus*, as well as parasitic infections, have been linked to both reduced food intake and alterations in gut microbial composition. These infection-driven disruptions may interfere with the gut–brain axis, potentially triggering or exacerbating EDs. Notably, epidemiological data suggest that up to 13.6% of individuals diagnosed with AN or BN report a viral infection during puberty or shortly before symptom onset [86]. Emerging evidence also underscores the relevance of gut microbial alterations in the onset and maintenance of AN and comorbid depression. Specific bacterial taxa such as *Coprococcus*, *Eggerthella*, and *Subdoligranulum* have been associated with mood dysregulation, likely through their effects on inflammatory cytokines that cross the blood–brain barrier and modulate neurotransmitter signaling [85]. Reduced microbial diversity and compositional shifts have been associated with heightened anxiety, depressive symptoms, and disordered eating behaviors, which reinforces the gut–brain axis as a potential therapeutic target. Recent longitudinal studies in hospitalized patients with AN have shown that the Firmicutes to Bacteroidetes (F:B) ratio tends to increase during treatment, although it often does not normalize to levels observed in healthy controls. In case-series analysis, individuals with chronic AN (>10 years duration) displayed persistently elevated F:B ratios, which suggests a distinct microbiome signature potentially associated with disease chronicity and poor treatment response [91].

Recent preclinical evidence also highlights the role of the gut microbiota in modulating food reward behavior and neuroinflammation, thereby possibly contributing to the dysregulation of hedonic feeding processes observed in EDs [82]. GABA can directly influence feeding behavior by suppressing the secretion of satiety hormones such as glucagon-like peptide-1 (GLP-1), peptide YY (PYY), and cholecystokinin (CCK). GABA-producing taxa, particularly from the genus Bacteroides, have been identified as key modulators of host appetite, acting through the downregulation of anorexigenic peptides and upregulation of orexigenic neuropeptides such as neuropeptide Y (NPY). This bidirectional activity counteracts appetite suppression and reveals a mechanistic link between microbial metabolism and neuroendocrine appetite regulation [92]. Moreover, dysregulation of appetite-related gut peptides such as ghrelin, CCK, and PYY has been observed in patients with AN, potentially contributing to diminished hunger, impaired satiety signaling, and gastrointestinal motility disturbances. Helal et al., in 2024, [91] observed that, despite significant increases in BMI during inpatient treatment, gut microbial composition exhibited limited responsiveness. The F:B ratio decreased after 4 weeks but increased again by week 12 in one patient, suggesting a delayed or nonlinear trajectory of microbiota normalization. This nonlinear recovery pattern underscores the complexity of microbiota restoration in AN and further supports the notion that gut microbial imbalances may exert persistent effects on host neurobiology. The gut microbiota and the CNS interact bidirectionally through the microbiota gut–brain axis, playing a significant role in the pathophysiology of various psychiatric disorders [88]. In this context, the potential to interrupt the progression of EDs by targeting the gut microbiota and its metabolites has garnered increasing interest among clinical researchers [93].

Psychotropic drugs, such as antidepressants and antipsychotics, which are widely used in individuals with EDs, may influence the composition and function of the gut microbial environment [94,95]. In parallel, emerging evidence in adolescent populations indicates that prolonged caloric restriction in AN can induce significant shifts in gut microbial diversity and composition. Several studies consistently report reduced α-diversity in patients with AN compared to healthy controls, along with notably elevated levels of *Methanobrevibacter smithii* and decreased levels of beneficial genera such as *Roseburia*, *Ruminococcus*, and *Clostridium* [75]. Furthermore, microbial profiles demonstrate notable reductions in genera such as *Romboutsia* and *Enterobacteriaceae*, along with increases in *Lachnospiraceae* and *Anaerostipes* [86]. The latter has been associated with clinical outcome predictions, independent of body weight at admission. 

Comparative studies between restrictive-type AN and binge–purging-type AN have revealed distinct microbial signatures. The binge–purging subtype is characterized by elevated levels of *Bifidobacterium*, *Bifidobacteriales*, and *Eubacteriaceae*, along with a reduced abundance of taxa such as *Haemophilus* and *Pasteurellaceae* [4]. By contrast, patients with the restrictive subtype exhibit elevated levels of *Clostridium coccoides*—a group implicated in the production of uremic toxins—and lower levels of *Odoribacter* and *Haemophilus* species [80]. According to a systematic review by Scala et al. [77], the gut microbiota composition in patients with AN showed significant alterations compared to controls; however, findings across studies remain inconsistent, reflecting heterogeneity in methodologies, patient populations, and clinical variables. The *Ruminococcaceae* family, within the phylum Firmicutes, was frequently reported to be altered in patients with AN compared to healthy controls, although findings remain discordant, with studies showing both increased and decreased relative abundance [78,89,96,97]. Similar inconsistencies have been noted for the genera *Clostridium* and *Romboutsia* [96,98]. These discrepancies may be attributable to methodological differences across studies. Although the participants were all female and of Caucasian ethnicity, variations in age, subtype of AN (restrictive vs. binge–purging), and psychotropic medication use could have influenced microbial composition. Additionally, differences in fecal sample collection procedures and DNA extraction protocols may also contribute to the observed variability. Meta-analyses have likewise identified variable but notable shifts in taxa such as *Alistipes*, *Parabacteroides*, *Turicibacter*, *Eisenbergiella*, and *Klebsiella*, which may help distinguish AN patients from controls. Species belonging to the Clostridium cluster (*Clostridium coccoides*, *Clostridium leptum*), are frequently reduced, while increases in *Enterobacteriaceae* and other Gram-negative taxa have been observed [82]. More consistent evidence points to a reduction in the abundance of gut-protective genera, including *Roseburia*, *Faecalibacterium*, *Ruminococcus*, and *Blautia*, all of which play critical roles in modulating inflammation and maintaining epithelial barrier integrity [78,79,89,97,99].

In a recent study by Shahid et al., individuals with comorbid MDD and AN exhibited significantly lower α-diversity than healthy controls, alongside elevated levels of *Blautia*, *Enterococcus*, and *Bifidobacterium*. These microbial patterns correlated with both C-reactive protein (CRP) levels and depression severity scores, which suggests their potential utility as biomarkers for MDD with comorbid AN [85].

*Bacteroides vulgatus* has been shown to alleviate anxiety-like behaviors and feeding abnormalities in animal models, indicating a potential protective role in the pathophysiology of AN [83]. By contrast, increased abundances of *Anaerostipes*, *Lactobacillus*, and *Methanobrevibacter smithii* have also been reported [89,96,98]. *Methanobrevibacter smithii* is considered to enhance metabolic efficiency during prolonged energy deficiency by converting CO_2_ to methane through hydrogen consumption, thereby promoting nutrient extraction under caloric restriction [76]. Although this adaptation may support energy conservation, methane production is known to impair gastrointestinal motility, potentially contributing to the high prevalence of constipation observed in AN [77]. Elevated levels are also linked to various metabolic and gastrointestinal conditions, including obesity, irritable bowel syndrome, non-alcoholic fatty liver disease, and cirrhosis [97,100]. In AN, its abundance negatively correlates with BMI, which further supports its role in host metabolic regulation [76].

According to an analysis by Yu et al. [88], *Peptostreptococcaceae*, *Coprococcus 3*, *Escherichia-Shigella*, *Lachnospiraceae* NC2004 group, and *Lachnospiraceae* UCG010 were identified as potential contributors to increasing AN risk. Conversely, four taxa, including *Cyanobacteria*, *Gammaproteobacteria*, *Mollicutes* RF9, and the *Eubacterium* brachy group, were associated with a reduced risk of AN. Mendelian Randomization (MR) analysis by Li et al. [83] revealed a causal relationship between specific microbial taxa and the risk of developing AN. Twelve taxa, including *Firmicutes E*, *RUG147*, *CAG-977*, *Desulfobacterota A*, and *Klebsiella pneumoniae*, were associated with a protective effect, while thirteen taxa, such as *ParaJ*, *Gillisia*, *Chlamydiales*, *Staphylococcus aureus*, and *Ruminococcus D*, were positively associated with AN risk, indicating a potential pathogenic role. Clinical treatment has been linked to increased detection of *Lactiplantibacillus* and *Bifidobacterium*, although control-matched data are lacking [81]. Elevated levels of *Bifidobacterium* were associated with successful weight gain, suggesting a supportive role in recovery. Hanachi et al. (2019) [101] reported decreased microbial richness and diversity in patients with severe AN undergoing enteral nutritional support. However, the continued underrepresentation of *Roseburia* post-refeeding reflects a long-term gut ecosystem disruption [79]. This microbial heterogeneity may be influenced by factors such as nutritional intake, BMI, hormonal status, physical activity, lifestyle habits, disease chronicity, and variability in stool sampling methods [77]. Notably, microbial alterations often persist despite weight restoration and psychological improvement [4,81,98], which supports the hypothesis that gut dysbiosis may represent a lasting trait in AN. Interestingly, animal studies have shown that fecal microbiota transplantation from high-feed-intake phenotypes can increase food intake in recipient animals, possibly via the microbial production of GABA. These findings suggest a causal role of gut microbiota in appetite regulation independent of nutritional status. Despite these findings supporting a potential causal role of gut microbiota in appetite regulation, translational relevance to humans remains to be fully established. Nevertheless, further research into microbiota-targeted interventions in the context of EDs merits attention [92].

### 3.2. Microbiota–Brain Interactions in Psychopathology

Emerging evidence highlights a multifaceted interplay between immune–inflammatory pathways, disordered eating behaviors, and mood-related psychopathology. Understanding these interconnections may provide novel insights into the etiological mechanisms underlying AN and its psychiatric comorbidities [102]. AN has been associated with a chronic low-grade inflammatory state, which may influence brain function and contribute to behavioral alterations [103].

However, recent longitudinal data from adolescent AN patients suggest a more complex and developmentally nuanced inflammatory profile. In contrast to adults, adolescents exhibit significantly decreased serum levels of interleukin-6 (IL-6) and interleukin-1β (IL-1β) at admission, which remain suppressed at discharge and one-year follow-up, while interleukin-15 (IL-15) levels are consistently elevated. These findings indicate age-specific immune regulation, potentially influenced by developmental stage or shorter illness duration. In addition, IL-15 levels are positively associated with *Romboutsia*, while an inverse association is observed between IL-15 and *Anaerostipes* and between tumor necrosis factor-α (TNF-α) and *Lachnospiraceae* abundance [84].

A hallmark of AN-related dysbiosis is the reduction in SCFA-producing genera with anti-inflammatory properties, particularly *Roseburia* and *Clostridium*. Decreased butyrate levels have been linked to increased intestinal permeability, facilitating the microbial translocation and systemic dissemination of microbial metabolites. This cascade can elevate pro-inflammatory cytokine levels and impact neuroimmune signaling [104]. In rodent models of EDs, neuroinflammatory changes are consistently observed: in AN models, elevated pro-inflammatory cytokines and the activation of astrocytes and microglia have been reported in the hippocampus; in BED, increased hypothalamic expression of inducible nitric oxide synthase (iNOS) has been noted; and in female mice, deletion of the IKKβ pathway in dopaminergic neurons reduced neuroinflammation and binge-like behaviors [82].

Inflammatory activity within the hypothalamus has been implicated in the induction of AN, largely through enhanced serotonin bioavailability and upregulated serotonergic signaling in this region [105]. Ghrelin, an orexigenic hormone commonly referred to as the “hunger signal”, plays a central role in appetite stimulation but is suppressed under inflammatory conditions, leading to early satiety and reduced food intake [106].

Pro-inflammatory cytokines may contribute to AN not only by inhibiting ghrelin synthesis but also by directly modulating CNS pathways that regulate energy balance. The lateral hypothalamus (LH), a key integrative hub for feeding and motivation, appears particularly sensitive to inflammatory signaling and is critical for regulating hunger and reward-based behaviors [107].

A recent study demonstrated that fluctuations in microbial taxa such as *Anaerostipes*, *Bacteroides*, *Faecalibacterium*, and *Lachnospiraceae* significantly correlated with changes in serum cytokine, particularly IL-15, IL-6, and IL-1β, which highlights the bidirectional nature of gut–immune–brain interactions in AN [84]. Commensal gut microorganisms participate in a broad range of neuroregulatory functions. They modulate the hypothalamic–pituitary–adrenal (HPA) axis and exert influence through both the vagus nerve and sympathetic pathways. Furthermore, the gut microbiota can produce or stimulate the release of several neuroactive compounds, including neurotransmitters and neuropeptides, through interactions with enteroendocrine cells [87]. The involvement of the bed nucleus of the stria terminalis (BNST) in appetite regulation is supported by evidence indicating that the oval region of the BNST can suppress feeding behavior through its projections to lateral hypothalamus (LH)-targeting subregions. Inflammatory signals have been shown to activate the BNST, which suggests that immune activation may influence feeding by modulating the BNST–LH microcircuit, potentially altering key neural substrates that govern hunger and satiety [108].

Recent findings also underscore the importance of dietary fiber in shaping gut microbiota composition. Fiber-rich diets that promote the proliferation of butyrate-producing genera such as *Roseburia* are associated with reduced levels of pro-inflammatory cytokines and improved clinical outcomes, including shorter treatment duration and lower severity of eating disorder psychopathology [77]. Additionally, specific chemokine receptors, particularly CXCR4, have been significantly associated with body composition parameters, including BMI and fat mass index, in individuals with AN [109]. These associations suggest that altered chemokine receptor expression on immune cells may contribute to the immunometabolic dysregulation underlying AN.

Ma et al. [87] identified distinct microbial signatures associated with various eating disorder risks. For example, *Clostridium* cluster I has been implicated as a potential risk factor in AN, while *Parasporobacterium* appears to exert a protective effect. Similarly, *Romboutsia* and *Eubacterium hallii* have been associated with increased and decreased susceptibility, respectively, in individuals with BN [87].

The ingestion of T-2 toxin, a trichothecene mycotoxin, has been consistently linked to anorexia in both humans and animal models, exerting profound adverse effects on overall health [110]. Once ingested, T-2 toxin disrupts the gut microbiota, leading to dysbiosis and impairing intestinal metabolic functions via mechanisms involving oxidative stress, inflammation, and apoptosis [110]. Notably, T-2 toxin is capable of crossing the blood–brain barrier and acting directly on the hypothalamus, where it stimulates the release of neurotransmitters such as 5-HT and substance P (SP) [111]. Elevated brain levels of 5-HT are associated with reduced food intake, while lower concentrations may result in hyperphagia and weight gain. Moreover, 5-HT is involved in modulating feeding circuits within the hypothalamus and dorsal raphe nucleus, influencing both satiety and the hedonic aspects of eating [112] Interestingly, certain microbial species such as *Faecalibacterium prausnitzii* have demonstrated a capacity to mitigate binge eating behavior in murine models by restoring kynurenic acid levels, a metabolite associated with satiety and glutamatergic modulation. A similar reduction *in F. prausnitzii* abundance has been observed in BN patients, which supports its potential translational relevance [82]. Concurrently, SP regulates appetite by activating neurokinin-1 (NK1) receptors, inducing nausea and vomiting and further exacerbating anorexic responses [113]. Additionally, exposure to T-2 toxin has been shown to elevate serum levels of gastrointestinal hormones, including CCK, gastric inhibitory peptide (GIP), GLP-1, and PYY, as well as pro-inflammatory cytokines, including IL-1β, IL-6, and TNF-α [114]. This systemic inflammatory response may disrupt gut–brain signaling pathways, thereby affecting appetite regulation. In murine models of T-2 toxin-induced anorexia, a significant increase in gut microbiota populations such as *Faecalibaculum* and *Allobaculum* is observed [115]. Such microbial shifts may represent a mechanistic link between T-2 toxin exposure, gut dysbiosis, and the intensification of anorectic signaling.

#### SCFAs, Appetite Regulation, and Metabolic Homeostasis

*Faecalibacterium*, *Bacteroides*, *Roseburia*, *Phascolarctobacterium*, and *Parabacteroides* are beneficial microbial genera commonly found in the human gastrointestinal tract. They are recognized for their ability to metabolize complex carbohydrates and produce SCFAs through fermentation, thereby contributing to gut health and host metabolic regulation [108]. SCFAs, including acetate, propionate, and butyrate, are key metabolic byproducts generated during the microbial fermentation of dietary fibers in the colon [116]. These compounds modulate host functions through immune, neuroendocrine, and humoral pathways [90]. They also play a pivotal role in gut–brain axis signaling by influencing appetite regulation via CNS circuits and hormonal cues. SCFAs bind to the G-protein-coupled free fatty acid receptor 2 (FFA2) and free fatty acid receptor 3 (FFA3) [117]. Studies in in vitro and in vivo mouse models and animals have shown that SCFAs, particularly propionate, bind to FFA3 at the level of adipose cells [118]. This activation leads to the production of leptin, the satiety hormone, at the level of the arcuate nucleus in the hypothalamus. FFA2 and FFA3 receptors are found in colon L cells that produce PYY and GLP-1; these reduce appetite [119]. PYY is released into the blood after meals, peaking after 1–2 h and remaining elevated for up to 6 h: intravenous infusions of PYY have been shown to reduce energy intake in both lean and obese subjects, acting through the inhibition of neuropeptide Y and the activation of POMC in the hypothalamus [120].

GLP-1 is an incretin, which stimulates insulin secretion and acts as a satiety signal: its blood levels increase after a meal, and intravenous infusions have been shown to reduce food intake in humans. GLP-1 receptors are present in many regions of the hypothalamus, contributing to the regulation of appetite. High consumption of fermentable dietary fiber increases the production of SCFAs in the colon, which, in turn, stimulates the release of PYY and GLP-1 and also increases the number of L cells in the colon; this leads to the reduction of subjective hunger [121,122].

**Figure 1:**
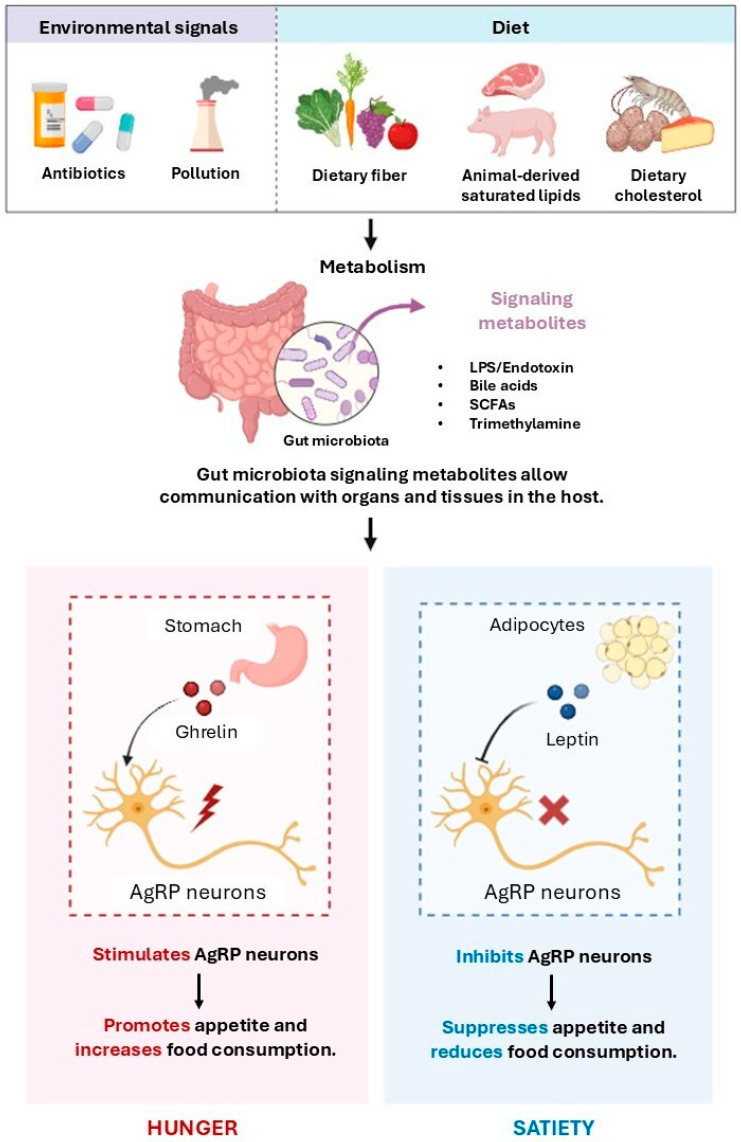
Gut microbiota signaling and appetite regulation.

Among the most compelling mechanisms by which SCFAs influence the CNS are their modulation of neuroinflammation, promotion of microglial maturation, and regulation of neurotransmitters and neurotrophic factor synthesis, including serotonin and brain-derived neurotrophic factor (BDNF) [123]. Peripherally, SCFAs help maintain intestinal homeostasis by reinforcing the epithelial barrier, enhancing mucus secretion, and exerting anti-inflammatory effects [108]. However, the impact of SCFAs on appetite remains an area of active debate, as findings across studies are not entirely consistent. For instance, Zhang et al. [124] reported an anorexigenic effect, noting enhanced secretion of satiety-related hormones from the colon. By contrast, Chen et al. [125] proposed that SCFAs may exert context-dependent effects on feeding behavior, with potential orexigenic outcomes influenced by dietary oligosaccharide content. These divergent findings suggest that the impact of SCFAs on appetite is shaped by a complex interplay of dietary and physiological conditions. Nonetheless, pro-inflammatory signals are more consistently associated with appetite suppression [108]. Figure 1 illustrates how the gut microbiota regulates appetite hormone signaling.

**Figure 2:**
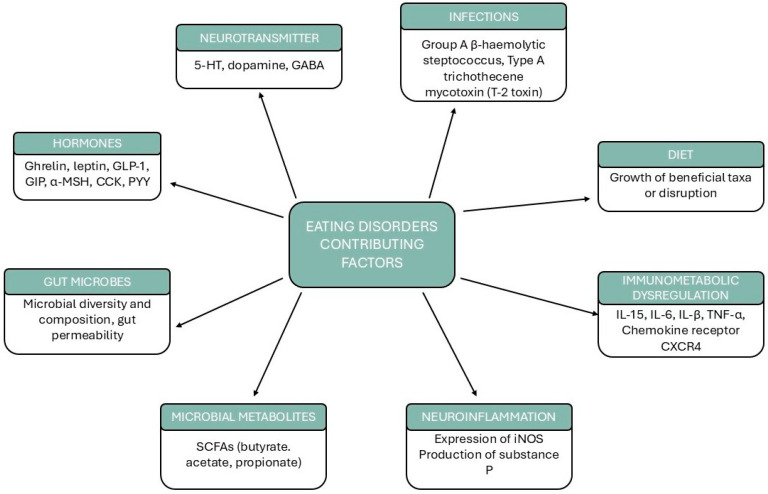
Multifaceted interplay of eating disorder contributing factors.

AN is frequently characterized by reduced insulin levels, largely as a consequence of chronic carbohydrate restriction. Under normal physiological conditions, *Roseburia inulinivorans* contributes to metabolic homeostasis by converting fucose into propionate, a SCFA that enhances insulin secretion via activation of the protein kinase C (PKC) signaling pathway [77]. In individuals with AN, the decreased abundance of *Roseburia* may impair this propionate-mediated insulinotropic mechanism, potentially exacerbating pre-existing endocrine dysfunction. Moreover, insufficient insulin availability may hinder the activation of insulin-sensitive dopaminergic receptors in the ventral tegmental area (VTA), a key brain region involved in reward processing and food-motivated behavior [126]. Although this mechanism remains partly hypothetical, impaired insulin signaling in these circuits may contribute to the mesocorticolimbic dysfunctions commonly reported in individuals with AN [127]. Alterations in the gut microbial community can interfere with fatty acid processing, potentially affecting the synthesis and catabolism of cholesterol and other lipid species. These disruptions in microbial composition and host lipid processing may influence the regulation of key neurotransmitters, including serotonin and dopamine, which are strongly implicated in the neuropsychiatric manifestations commonly observed in AN [83]. Severe caloric restriction in AN results in profound nutritional deficiencies, which trigger a compensatory hepatic response marked by the enhanced β-oxidation of fatty acids to meet energy demands [128]. This metabolic adaptation leads to increased production of ketone bodies and elevated levels of circulating free fatty acids, contributing to a systemic state of metabolic stress. As a result, energy homeostasis is disrupted, and the body composition is significantly altered, which further reinforces the physiological consequences of chronic undernutrition. Figure 2 attempts to summarize the aspects discussed so far that are implicated in the pathophysiology of EDs.

## 4. Specific Considerations in Women with EDs

### 4.1. Estrogen and Progesterone Influences on Microbiota: Menstrual Cycle, Pregnancy, and Menopause and Microbial Shifts in Women

Female sex hormones, particularly estrogen and progesterone, have a profound influence on the intestinal and vaginal microbiota and, in turn, can be regulated by the latter, modulating the systemic bioavailability of the hormones themselves and, thus, influencing endocrine pathophysiology and host responses [129,130,131]. This bi-directional dialogue is mediated in the intestine by the estrogenome, bacterial genes involved in the deconjugation of estrogens through the enzyme β-glucuronidase: this mechanism determines the recirculation of estrogens to the systemic level after hepatic passage, varying their bioavailability and systemic efficacy [5,132]. High levels of estrogen metabolites are linked to microbial diversity, whereas high levels of unmetabolized parent estrogens are linked to breast cancer development [64]. The ratio of estrogen metabolites to parent estrogens may hold potential as a future biomarker, providing valuable information for understanding and managing estrogen-related conditions; however, its diagnostic utility remains to be established. The microbiota, therefore, acts as an endocrine system, producing and metabolizing hormone-like compounds that influence sex hormones. The expression of estrogen receptor β (ERβ) and serum concentrations of steroid hormones, particularly estradiol (E2), fluctuate throughout life, underlining the importance of estrogen regulation for a woman’s overall health. In the intestine, estrogen regulates motility, permeability, and mucus composition, forming a favorable environment for mutualistic bacteria. E2 promotes the diversity of the gut microbiota, promoting the growth of beneficial bacteria such as *Lactobacillus* and *Bifidobacterium*, known for their anti-inflammatory, immunomodulatory, and protective properties toward the intestinal barrier [133,134]. *Prevotella intermedius* utilizes E2 and progesterone for its growth [135], *Costridium scindens* converts glucocorticoids into androgens [136], and antibiotics reduce estrogen concentrations. The gut microbiota plays an important part in estrogen metabolism. In the vagina, high levels of estrogen during the fertile phase lower the vaginal pH, favoring the prevalence of *Lactobacillus* spp. [137]. The predominance of *Lactobacillus* species in a balanced vaginal microbiota is largely attributable to their ability to produce antimicrobial compounds. These include lactic acid, which helps to maintain an acidic vaginal environment unfavorable to the growth of pathogenic bacteria; bacteriocins, which are narrow-spectrum antimicrobial peptides that selectively target harmful bacteria; and hydrogen peroxide (H_2_O_2_), which plays a crucial role in the host’s defense against infection [138]. These antimicrobial substances synergistically contribute to maintaining the stability and resilience of the vaginal microbiota, which highlights the importance of lactobacillus dominance in maintaining vaginal health [139,140]. This dominance helps prevent vaginal infections, may reduce the risk of sexually transmitted infections (STIs), and promotes better reproductive health and more effective gynecological treatments [141]. An imbalance, with a reduction in *Lactobacilli*, can compromise these mechanisms [142]. The cyclical fluctuations of estrogen and progesterone during the menstrual cycle, as well as the more stable hormonal changes that occur during pregnancy and menopause, affect the balance of the microbial ecosystem and result in changes in the composition and stability of the gut microbiota [143,144]. These changes may be accompanied by mood changes, appetite alterations, and food cravings, which are also common in eating disorders [145]. However, these changes may be maladaptive in the presence of preexisting psychological vulnerabilities, promoting the alteration of the gut–brain axis and contributing to the development or exacerbation of dysfunctional eating symptoms, especially in the presence of depressive disorders or gestational anxiety [146]. The use of hormonal contraceptives can also affect the microbiota, stabilizing the vaginal population depending on the type of hormone replacement used [147,148,149]. Alterations in the microbiota, which can reduce estrobolome activity, result in the increased fecal excretion of estrogen, resulting in relative hypoestrogenism; conversely, if they produce β-glucuronidase and dehydrogenase, enzymes that convert inactive estrogens back to active forms, they increase their systemic bioavailability, with implications for the pathogenesis of estrogen-sensitive and hormone-related pathologies such as premenstrual syndrome, endometriosis, infertility, polycystic ovary syndrome (PCOS), estrogen-dependent cancers, obesity, cardiovascular disease, and osteoporosis [150,151,152,153,154,155,156,157]. Alterations in the composition of the gut microbiota have been observed in these diseases, which suggests a potential role of the microbiota in their development [155].

The appearance of these early signs of menopause corresponds to a decline in hormone production by the ovary: E2 declines at the expense of estrone (E1), which is the main estrogen in the climacteric phase. FSH increases rapidly, while LH decreases slowly, reversing the FSH/LH ratio [158]. Decreased ovarian-derived estrogen production is associated with numerous physical changes related to medium- and long-term target organ metabolic changes: uro-genital atrophy, resulting in vaginal and urinary tract inflammation; osteoporosis; degenerative arthropathy; angina pectoris; atheromatosis; and thrombosis. The vaginal microbiota shows a decrease in Lactobacilli, which enhances the susceptibility to infection, and the gut microbiota tends toward a less diverse and more pro-inflammatory composition [159]. This suggests that the gut microbiota may play an essential role in the regulation of estrogen levels and metabolism during menopause. Such changes potentiate the risk of metabolic disorders, weight gain, and a worsening systemic inflammatory pattern [160]. In addition, the gut microbiota can metabolize estrogen-like compounds found in foods, such as soy isoflavones, which, in turn, promote the growth of beneficial bacteria [144].

Functional Hypothalamic Amenorrhea (FHA) is the absence of menstruation due to low weight, excessive exercise, and/or stress. FHA occurs when energy reserves do not meet the body’s needs, leading to the decreased secretion of gonadotropin-releasing hormone (GnRH) from the hypothalamus [161]. This, in turn, reduces FSH and LH hormones, impairing follicular development and estrogen secretion. Stress, by increasing cortisol levels, also inhibits GnRH secretion, which explains menstrual irregularity during periods of high stress. The relationship between stress and FHA is bidirectional: stress can trigger the suppression of the hypothalamic–pituitary–ovarian (HPO) axis, and, conversely, low estrogen levels greatly affect neuropsychological status, thus creating a vicious cycle. Amenorrheic women—thus, with hypoestrogenism—have greater increases in heart rate, blood pressure, and cortisol levels in response to psychological stress: low levels of gonadal hormones, oxytocin, and leptin and high levels of cortisol and PYY have been implicated in the psychopathology of eating disorders and the symptoms of anxiety and depression in AN [162]. Misra et al. saw that the administration of transdermal E2 reduced trait anxiety in girls with AN and prevented the increase in state anxiety observed with weight gain over time compared with those receiving placebo, which suggests a role of estrogen modulation [163]. Some studies have confirmed cognitive dysfunction in adolescents and adult women with FHA and hypoestrogenism, with improvement in these measures with the resumption of menstruation or estrogen administration [164,165]. Women with FHA exhibit greater perfectionism and attention to others’ judgment, as well as greater difficulty in coping with stress, than women with regular cycles [166]. Transdermal E2 replacement therapy in adolescent girls with AN has been shown to prevent the increase in body dissatisfaction that occurs with weight gain over time compared to those who remain hypoestrogenic [167].

The sharp estrogen decline of menopause is accompanied by a reduction in microbial diversity and a predominance of pathogenic or opportunistic species in both the gut and vagina: this dysbiosis may contribute to metabolic disorders, increased intestinal permeability, and a chronic low-grade inflammatory state [168]. Such changes have also been linked to depressive symptoms, appetite alterations, and emotional dysregulation, which may provide a fertile ground for the late onset or persistence of eating disorders [169]. Alterations in the microbiota associated with phases of the female hormonal cycle may amplify psychological vulnerability in individuals predisposed to eating disorders [170]. In particular, dysbiosis may modulate the gut–brain axis, affecting neurotransmitter production (such as serotonin, GABA, and dopamine), hypothalamic–gut axis activity, and stress response [171]. Hormonal changes may exacerbate symptoms of body image dissatisfaction, binge eating, restriction, or compensatory behaviors [172]. Puberty, pregnancy, and menopause pose a risk for the exacerbation of eating disorders due to the perceived microbial and body changes [173]. AN occurs 9 times out of 10 in the female sex, and the onset is often at pubertal age, which suggests the involvement of ovarian hormones in the genesis of the condition [174]. Estrogen has a depressant effect on weight, so it has been hypothesized that increased estrogen at pubertal age may promote the onset of AN [175]. Women with PCOS, on the other hand, due to increased steroid hormones, may experience a stimulatory effect on body weight and appetite, with an increased risk of developing bulimia and an uncontrolled eating disorder [176]. Recent studies have shown that menstrual cycle phases appear to influence binge eating episodes [176,177]. Therefore, there is a need to evaluate the microbiota as an integral part of a psychoneuroendocrine view of the treatment and prevention of eating disorders in women, with particular attention to periods of hormonal vulnerability.

Progesterone exerts significant effects on the microbiota through its anti-inflammatory action and immune modulation [178,179]. Its cyclic fluctuations influence intestinal motility and, together with estrogen, contribute to the lowering of the vaginal pH, promoting the growth of *Lactobacillus* spp. [180]; in addition, some strains such as *Streptococcus mutans* can metabolize progesterone [181,182]. During pregnancy, the stabilization of high levels of progesterone is associated with significant remodeling of the gut microbiota, with an increase in bacteria being involved in more efficient energy metabolism and adaptive homeostasis conducive to gestation, a reduction in microbial diversity, and a tendency toward a more inflammatory and obesogenic setup functional for maternal–fetal energy requirements [183,184,185,186]. In parallel, the vaginal microbiota consolidates into a *Lactobacillus*-dominated configuration, promoting a protective barrier against pathogens and contributing to fetal health [187]. An endometrial microbiota, on the other hand, not dominated by *Lactobacillus* is associated with reduced rates of implantation, pregnancy, and live births, although the specific mechanisms by which the bacteria interfere with embryo implantation are not yet fully understood. The dysbiotic endometrial microbiota could change key inflammatory pathways necessary for successful embryo implantation and pregnancy development [188]. These changes can affect maternal and fetal health and have been associated with risks such as gestational diabetes and preterm delivery [189,190]. The microbiota–sex hormone relationship, therefore, may influence reproductive outcome. Pregnancy induces a profound restructuring of the gut microbiota, with an increase in pro-inflammatory bacteria, such as Proteobacteria and Actinobacteria, which are able to degrade steroids and contribute to more efficient energy metabolism and controlled inflammation, which is useful for fetal growth support. Progesterone, the predominant hormone in pregnancy, influences the microbiota, favoring an increase in Bifidobacterium [191]. Women who have preterm delivery often have lower levels of *Lactobacillus crispatus* in the vaginal microbiota and higher levels of *Sneathia amnii.* These alterations are associated with increased pro-inflammatory cytokines in vaginal fluid [192]. Furthermore, the maternal vaginal microbiome has been shown to influence the neonatal gut microbiome; in fact, those born via cesarean section have significantly reduced microbial diversity compared to those born vaginally [193].

The menstrual cycle, pregnancy, and menopause, therefore, represent three phases of the female biological continuum, characterized by endocrine changes, which exert a systemic impact on metabolism, immune function, mood regulation, and microbiota composition. The hormonal fluctuations that accompany these phases affect not only the reproductive systems but also the microbial ecosystem, contributing to a complex bidirectional interaction between hormones, gut microbiota, and feeding behavior [3]. In light of these interactions, the microbiota–gut–brain axis emerges as a key element in the pathogenesis and modulation of eating disorders.

### 4.2. Diet

Soy isoflavone supplementation has been shown to increase *Bifidobacterium* concentrations and suppress the growth of unclassified *Clostridiaceae* in postmenopausal women [64]. In this context, *Bifidobacterium* improves nutrient absorption, supports immune function, and prevents infection, while *Clostridiaceae* are associated with inflammation and obesity. The intake of total and saturated fats in large amounts has a negative effect on the composition of the gut microbiota: in these cases, the Firmicutes–Bacteroidetes (F:B) ratio increases, affecting the microbial abundance and density. The estrogen–microbiota ratio influences obesity: the microbiota metabolizes estrogen-like compounds, phytoestrogens, which can stimulate ERβ. Diets rich in phytoestrogens are associated with weight gain, whereas lignan metabolites (natural phytoestrogens) are associated with reduced obesity, reduced LDL cholesterol, and glycemic balance, which highlights the beneficial activating role of bacteria. Vitamins act as enzyme cofactors, and because they are not synthesized by the body (apart from some produced by gut bacteria), they must be taken through the diet [194]. Minerals actively interact with the gut microbiota: for example, high calcium intake can influence the lean phenotype by increasing specific bacterial species [195]. Proper nutrition is also a pillar of the proper functioning of the hypothalamic–ovarian axis, which regulates estrogen production during puberty. A balanced diet consists of plenty of fruits, vegetables, lean protein, and whole grains, while limiting added sugars, sodium, and saturated fat. When the body does not receive sufficient energy and nutrition, maturation and growth during adolescence are impaired, mainly due to hypothalamic suppression. Healthy lifestyle habits in perinatality are believed to be a guarantee for successful pregnancies and the prevention of gestational diseases. Healthy eating, weight management, physical activity, planned pregnancy, and physical, mental, and psychosocial health are part of prenatal wellness, as established by the World Health Organization. Modulation of the microbiome, behavioral strategies such as smoking cessation, anxiety and stress reduction, sleep quality, maternal genetics, age, social class, and education can influence maternal quality of life, helping to maintain a healthy pregnancy and reduce the risk of complications [196]. An unbalanced diet can lead to microbiota imbalance, increase intestinal permeability, and affect insulin and androgen levels [197,198].

## 5. Potential Microbiota-Targeted Interventions

The ongoing management of eating disorders remains highly challenging, as conventional treatment strategies often show only modest success, which underscores the necessity of novel therapeutic approaches to enhance clinical outcomes. The complex mechanisms underlying the development of EDs and the difficult management of those conditions have led researchers to focus interest on novel approaches taking into consideration blood biomarkers, neuroimaging, and markers of gut–brain interaction. These developments have fostered the testing of innovative biological therapies, including neuromodulation techniques (transcranial magnetic stimulation (TMS), transcranial Direct Current Stimulation (tDCS), deep brain stimulation (DBS)), psychotropic drugs (such as ketamine, psilocybin, metreleptin, and GLP-1 agonists), and combined approaches with specific diets and microbiome-based treatments [198].

### 5.1. Prebiotics, Probiotics, and Dietary Modification Strategies

Recent attention is emerging on potential interventions addressing gut microbiota dysbiosis as a new frontier for the treatment of such disorders. The therapeutic benefits of integrating prebiotics and probiotics as an adjunctive therapy in mood disorders, especially major depression, and anxiety disorders in order to achieve intestinal homeostasis have been established in the literature [6,199,200,201]; therefore, more interest has grown in the use of such strategies in anorexia nervosa, bulimia, binge eating disorder, and other related conditions as well.

In 2013, the International Scientific Association for Probiotics and Prebiotics (ISAPP) preserved the definition of probiotic provided by the FAO/OMS in 2001, with a slight grammar correction: “live microorganisms that, when administered in adequate amounts, confer a health benefit on the host” [202]. Later, Dinan et al. defined psychobiotics as a category of probiotics with potential positive effects on mental health symptoms through the gut–brain axis interaction [203].

The systematic review by Bahari et al. [204] in 2024 analyzed twelve human and animal studies in order to explore the potential impact of probiotics on EDs. Preliminary results, despite the small number of available studies, suggest that probiotic supplementation may exert beneficial effects on eating behavior, in particular, by improving satiety, attenuating compulsive eating patterns, and modulating food addiction. In certain cases, an impact on the hormonal regulation of appetite, the modification of compulsive behavior, and anthropometric parameters related to body weight was observed. Evidence also indicates that probiotics could interact with the microbiota–gut–brain axis by influencing the gut microbial composition and the production of metabolites [205], hormones, and neurotransmitters and by modulating the immune system, with potential benefits also on mood, anxiety, and stress, factors closely linked to dysregulated eating behaviors [204]. In Nicol et al.’s study, C57Bl6 mice subjected to chronic mild stress conditions were supplemented with a mix of *Lactobacillus salivarius* LS7892, now called *Ligilactobacillus salivarius,* and *Lactobacillus gasseri* (LG) LG6410 strains compared to a placebo group. The results from the experiment showed a reduction in sucrose intake and an increase in water, the mitigation of stress-induced sugar craving, and the amelioration of mild depressive symptoms in the probiotic group [206]. Despite the use of only male mice in the study, the results provide worthwhile insights and merit further analysis in humans. Trinh et al. (2023) [207], on the other hand, investigated how chronic food restriction, refeeding, and probiotic supplementation affect the gut permeability and the immune-related lymphoid tissue growth using an activity-based anorexia (ABA) rat model. Limited food intake, combined with excessive activity, causes damage to the gut barrier and stimulates the gut-associated lymphoid tissue (GALT), markers of increased inflammation and intestinal stress. Remarkably, a mixture of eight different bacterial species (VSL#3^®^ supplementation), even without weight gain, helped reduce immune activity in the gut and showed signs of healing the gut barrier (Table 2). This points to a promising, non-invasive way to support gut health. Furthermore, still using an ABA model, Verspohl et al. [208] observed how a chronic starvation condition in female rats led to a depletion of glial cells (especially astrocytes, while a limited reduction in oligodendrocytes). This depletion appeared to be linked to reduced cell proliferation, possibly reflecting a neuroinflammatory environment that contributes to neurodegeneration, which suggests that neuroinflammation plays a role in the pathophysiology of AN. In the study, supplementation with omega-3 fatty acids (FAs) reduced running wheel activity, which suggests their potential use in the diet as a strategy to support weight gain. A slight reduction in hyperactivity was shown in the probiotic supplementation group; however, the difference was not significant. Eventually, neither omega-3 FAs nor probiotics restored glial cells, possibly due to limitations in dosage, intervention duration, or effect size [208]. Liu et al. [205] used an in vitro three-stage colonic model to simulate the human gut under dietary conditions mimicking AN. The results obtained from the study highlighted a substantial decline in all bacterial groups, with a reduction in beneficial microbial taxa (e.g., *Bifidobacterium*, *Lactobacillus, Roseburia*, and *Faecalibacterium prausnitzii*) and microbial metabolites such as SCFAs and neurotransmitters (5-HT, DA, and GABA). Supplementation with the prebiotic fructo-oligosaccharides (FOS) or the probiotic yeast *Saccharomyces boulardii* (5 × 108 cfu) partially restored microbial diversity and abundance, elevated levels of SCFAs (e.g., acetate, propionate, and butyrate), and increased neurotransmitter production, particularly 5-HT and GABA, which validates the potential use of psychobiotics as an adjunctive therapy for AN. Despite the promising results, an in vitro model hardly replicates the complex characteristics of in vivo conditions, not only regarding the gut microbial community and metabolites but also considering the interconnection with the brain with its own functionalities [205].

Despite optimistic results from animal studies, human evidence is still scarce and evolving. Gröbner et al. [209] are carrying out a study protocol to evaluate the efficacy of supplementation with multistrain probiotics containing *Lactobacillus* and *Bifidobacterium* species in adolescents with anorexia nervosa, alongside traditional pharmacotherapy. The aim of the study is to assess whether the administration of probiotics for 6 months in adolescents between 13 and 19 years of age will lead to changes in the intestinal microbiota, symptoms related to the psychopathology of eating disorders, changes in gastrointestinal symptoms, weight gain, or changes in inflammation markers (Table 3). There is no mention of sex-specific microbial biomarkers or the inclusion of female-only patients [209]. In parallel, emerging evidence from related clinical populations further supports the gut–brain axis as a therapeutic target. A randomized clinical trial by Ghafouri-Taleghani et al. [210] investigated the effects of probiotic supplementation in patients with food addiction and weight regain after metabolic bariatric surgery. Fifty patients were divided into two groups: the intervention group, receiving probiotics for 12 weeks + cognitive behavioral therapy (CBT) + a weight loss program; and the placebo group, undergoing CBT + a weight loss program other than placebo. At the end of the trial, the patients in the intervention group, composed mainly of women, exhibited greater improvements in anthropometric measures, food addiction scores, and eating behavior, particularly in uncontrolled eating and cognitive restraint, compared to those receiving CBT and diet alone. Behavioral changes were accompanied by a significant increase in oxytocin and a reduction in leptin, suggesting a modulation of appetite-regulating hormones, likely mediated through the microbiota–gut–brain axis [210]. Although their study focused on a post-obesity treatment population, its findings suggest shared pathways relevant to restrictive disorders like anorexia nervosa. The dysregulation of satiety hormones and emotional regulation, which is a common element in both disorders, may be influenced by microbial composition and function. Furthermore, the observed correlations between oxytocin/leptin and eating behavior corroborate the hypothesis that probiotics may serve as modulators of both physiological and psychological aspects of eating disorders. In addition, Ghafouri-Taleghani et al.’s study does not examine plausible differences in participants’ gut microbiota based on specific bariatric surgery previously received, while evidence in the literature is paying attention to distinct alterations in gut microbial composition and metabolite production [211,212]. In Komorniak et al.’s study, patients who underwent Roux-en-Y gastric bypass (RYGB) showed an increased presence of *Veillonella* and *Roseburia*, while those receiving sleeve gastrectomy (SG) exhibited greater levels of *Christensenellaceae R-7 group*, including *Subdoligranulum*, *Oscillibacter*, and *UCG-005*. Moreover, the probiotics’ assumption was not effective on the mood symptoms of the participants, which suggests that the dietary changes were the primary driver of the mental health improvements [211].

While neither study isolated the effects of probiotics alone, the majority support their use as adjunctive strategies within comprehensive treatment programs. Choi et al.’s study [6], on the contrary, demonstrated how *Lacticaseibacillus rhamnosus* HA-114 supplementation ameliorated binge eating behaviors, mood symptoms, and metabolic indices in adults with overweight, along with following a dietary intervention of weight loss.

However, due to the small amount of evidence, the methodological heterogeneity, and limited sample sizes, it is still difficult to draw robust conclusions on the use of psychobiotics for eating disorders [204].

### 5.2. Fecal Microbiota Transplantation (FMT): Current Evidence

We have seen so far how maintaining a gut microbiota homeostasis is essential for the proper functioning of the gut–brain axis and how dysbiosis can be a part of the pathophysiology of EDs [170,213]. Therefore, restoring the microbial balance represents a promising therapeutic target in the treatment of EDs, especially through the use of FMT. It has already been established in the literature how fecal transplants from healthy donors reduced depressive and anxiety-like symptoms [214]. Given the tight connection between altered gut function and the brain in the pathophysiology of AN and other eating disorders, FMT could be a useful strategy in eating disorder treatment, possibly reducing time of recovery.

The case report by de Clercq et al. [215] presents the first documented case of weight gain after FMT in a 26-year-old anorexic patient in a state of recurrent underweight. Despite good adherence to a stable high-calorie diet, the patient maintained a low body weight after discharge from a recovery in hospital. After an FMT procedure from a healthy donor, their weight was monitored at three time points, registering an increment of 6.3 kg at 36 weeks and a 55% increase in body fat, without significant changes in diet. This result was accompanied by a growing gut microbial diversity, particularly the enrichment of *Akkermansia muciniphila* and high levels of SCFAs such as acetate and butyrate. Their study, despite limitations related to the single-patient design, lack of physical activity monitoring, and concomitant immunosuppressive therapy for an autoimmune dermatomyositis, showed a promising result, laying the foundation for further studies [215]. Wilson et al. designed an open-label pilot study involving 20 females with anorexia nervosa, aged 16–32 years. The primary outcome is an evaluation of the gut microbiome composition at 3 weeks post-FMT, while secondary outcomes are planned to measure body composition and eating disorder, depression, and anxiety symptoms and to assess the feasibility of the treatment [216].

Animal studies, on the contrary, show mixed results. Research by Maschek et al. in 2025 [217] examined antibiotic-treated mice transplanted with gut microbiota from AN or control donors. The study found that mice receiving AN microbiota showed significantly reduced food intake and increased serum levels of satiety hormones, such as PYY and leptin, despite the absence of weight variation. Behavioral testing revealed decreased locomotor activity in AN-colonized mice, which may explain the unchanged body mass. Eventually, a second FMT from non-eating disorder donors partly ameliorated these effects, which suggests that AN-associated gut microbiota can modulate appetite regulation and behavior via the gut–brain axis and that FMT from healthy donors may balance these alterations and offer therapeutic potential for AN [217]. However, recent findings showed that FMT from adolescent patients with AN did not alter cognitive flexibility, anxiety-like behavior, or dopamine signaling in rats. These findings indicate that this approach remains an emerging area of research requiring further investigation and validation. The available evidence is still limited and based on studies with small sample sizes. The efficacy of microbiota transfer may be constrained by low transplant efficiency, which highlights the need for standardized and optimized protocols in future studies [218].

### 5.3. Precision Nutrition, Personalized Microbiome-Based Interventions for Women, and Sex-Specific Microbial Biomarkers

Emerging research emphasizes the relevance of precision nutrition and personalized microbiome-based interventions in addressing EDs [219], especially given their high prevalence in women [220].

Precision nutrition approaches tailored to the specific needs of single patients, including dietary plans and psychobiotic supplementation customized to individuals’ microbiota and nutritional profile, are crucial for restoring a healthy gut and improving outcomes. Specific bacterial taxa and their interactions with key metabolites are altered in eating disorder patients, and many of these relationships shift direction depending on the nutritional state. This suggests not only a structural but also a functional reprogramming of the gut microbiome in response to the disease state [221].

Kan et al. [219] remark upon the importance of the “3 P’s” in AN, which incorporate the predisposing, precipitating, and perpetuating factors. Precipitating factors include weight loss, dieting, stresses, and life events, while perpetuating factors take into consideration the neuroadaptation and neuroprogression processes and environmental factors, like social exclusion [219]. It has been reported in the literature how women face different gender-related social determinants of health, which could contribute to the development of different pathogenic profiles [222] over the presence of possible sex-based metabolic structural differences [223]. Moreover, in modern society, idealized appearance content and easy access to unhealthy lifestyles via social media contribute to a high-standard body image and disordered eating [224], primarily affecting women.

To fully understand the complex mechanisms underlying EDs and their influence on host physiology, microbiome profiling and integrated omics approaches are essential tools in modern biomedical research. Multi-omics profiling allows researchers to identify biomarkers and potential therapeutic targets. Sex-specific microbial biomarkers are emerging as potential contributors to EDs [225]. For example, Tennoune et al.’s study [226] highlighted how *E. coli* K12 was present in the gut of female rats and not in males before supplementation. *E. coli* produces caseinolytic protease B (ClpB), a protein mimicking the anorexigenic neuropeptide α-MSH. Once administered, *E. coli* led to increased food intake and weight gain in females, whereas males showed a different immune response. This suggests that the gut–brain axis may operate differently by sex, potentially contributing to the higher ED prevalence in women. Nevertheless, these findings warrant validation in human studies [226].

In conclusion, the association of precision nutrition and microbiome-based treatments holds promise for more effective, unique therapies for women with EDs. Preliminary findings are encouraging, although robust clinical evidence is still limited. Further research is needed to develop and validate these personalized interventions.

## 6. Conclusions

This narrative review contributes to outlining the complex interactions between microbial alterations and the pathophysiology of EDs, with a special focus on women and AN, emphasizing both biological mechanisms and therapeutic implications.

Noteworthy evidence suggests that gut microbiota alterations are not merely secondary effects of disordered eating but rather may actively contribute to the onset, maintenance, and relapse of EDs in women. Although preclinical studies support the use of probiotics and microbial modulation in alleviating symptoms, clinical applications remain preliminary.

Existing research often derives from animal models that do not fully replicate the complexity of physiological and behavioral responses, especially in female-specific contexts of EDs. In addition, the use of male mice may not adequately capture the mechanisms underlying in EDs, which are significantly more prevalent in the female population. This highlights the need for improved translational models and caution in extrapolating preclinical findings.

Future research must address key gaps through longitudinal, multi-center trials that examine microbial signatures in relation to illness duration, subtype, diet history, and behavioral patterns. Personalized interventions, such as strain-specific probiotics or microbiota-informed dietary plans, may enhance standard treatments and obtain encouraging outcomes. Notably, most of the limited evidence present in the literature derives from multi-strain probiotics formulation. Research should also investigate deeper on fecal microbiota transplantation, which remains an emerging yet limited approach. It would be necessary, in the future, to include a larger study cohort and to pay attention to the optimal microbiota donor for individualized cases. As our understanding deepens, targeting the gut microbiota represents a promising target for precision interventions in women with eating disorders.

## Figures and Tables

**Table 1:** Overview of gut microbiota taxa and their features in anorexia nervosa.

Studies	Microbial Taxa	Feature	Mechanistic Insights
Zimmermann-Rösner & Prehn-Kristensen, 2024 [75]; Zhao et al., 2024 [76]; Scala et al., 2025 [77].	*Methanobrevibacter smithii*	Strongly ↑ in AN; microbial adaptation to caloric restriction: converts CO_2_ into methane using H_2_, enhancing energy extraction. Methane slows intestinal motility, contributing to constipation. Negatively correlated with BMI.	CO_2_ conversion into methane via hydrogen; ↑ energy efficiency; methane slows gut motility → constipation; negatively correlated with BMI.
Prochazkova et al., 2021 [78]; Mondot et al., 2022 [79].	*Roseburia*	Butyrate-producing genus with anti-inflammatory properties; enhances epithelial barrier integrity and modulates local immunity. ↓ in AN even post-refeeding.	Fermentation of dietary fibers → butyrate production; activation of Tregs; enhancement of epithelial barrier and modulation of anti-inflammatory cytokines.
Zimmermann-Rösner & Prehn-Kristensen, 2024 [75].	*Ruminococcus*	Involved in fiber degradation and SCFA production. Reduced in AN; associated with dysbiosis and mucosal alteration.	Degradation of complex polysaccharides → SCFAs; supports mucosal integrity and local immune regulation.
Baenas et al., 2024 [80].	*Clostridium coccoides*	↑ in restrictive-type AN; implicated in uremic toxin production and potential intestinal damage.	Uremic toxin production; contributes to pro-inflammatory gut dysbiosis.
Monteleone et al., 2021 [4]; Morisaki et al., 2023 [81].	*Bifidobacterium*	↑ in binge–purging subtype; higher levels linked to weight recovery. Supports immunity and regulates inflammation.	Carbohydrate metabolism and lactic acid production; promotes growth of beneficial microbes; stimulates tolerogenic dendritic cells.
Huwart et al., 2025 [82].	*Faecalibacterium prausnitzii*	Butyrate producer; associated with anti-inflammatory effects. ↓ in AN and BN; alleviates binge-like behavior in mice.	Butyrate production → activation of GPR43 and GPR109A receptors; NF-κB inhibition; IL-6 and TNF-α suppression; protection against dysbiosis.
Li Z et al., 2025 [83].	*Bacteroides vulgatus*	Modulates behavior; ↓ anxiety and disordered eating in animal models.	GABAergic signaling modulation; reduction in anxiety-related neuronal activity (amygdala); influences HPA axis function.
Käver et al., 2024 [84].	*Anaerostipes*	↑ in AN; negatively associated with IL-15. Influences immune signaling and inflammation.	Fermentation of complex sugars → SCFAs; IL-15 inhibition; potential interaction with TLR pathways.
Shahid et al., 2025 [85].	*Coprococcus*	Involved in mood regulation via cytokine and neurotransmitter signaling. ↓ in AN with comorbid depression.	Inflammatory regulation via SCFAs and neurotransmitter synthesis; interaction with gut–brain axis pathways.
Bozzola et al., 2024 [86]; Käver et al., 2024 [84].	*Lachnospiraceae*	↑ in AN; inversely related to TNF-α; predicts clinical outcomes and gut function.	SCFA production; interaction with TLR and PRR; inverse correlation with TNF-α and inflammatory biomarkers.
Bozzola et al., 2024 [86]; Huwart et al., 2025 [82].	*Enterobacteriaceae*	↓ in acute AN, ↑ in chronic forms. Gram-negative; LPS-linked inflammation.	Gram-negative component; lipopolysaccharide (LPS) stimulates immune response; linked to chronic inflammation.
Bozzola et al., 2024 [86]; Käver et al., 2024 [84].	*Romboutsia*	↓ in AN; positively correlates with IL-15; relevant to gut–brain axis regulation.	Modulates IL-15 levels; possibly activates JAK/STAT pathways in mucosal immune responses.
Ma et al., 2025 [87]; Huwart et al., 2025 [82]	*Clostridium cluster*	Associated with AN risk; involved in appetite and neuroactive metabolite regulation and neuroinflammation.	Production of neuroactive metabolites (e.g., tryptophan derivatives); modulation of serotonergic receptors. Fermentation of dietary fibers; SCFA production.
Ma et al., 2025 [87].	*Eubacterium hallii*	SCFA producer; ↓ in EDs, especially bulimia.	Fermentation of polysaccharides; propionate production; PPAR-γ stimulation and intestinal inflammation reduction.
Yu et al., 2024 [88].	*Peptostreptococcaceae*	Potential risk factor for AN from MR studies; affects innate immune signaling.	Expression of microbial antigens activating innate immunity; possible effects on mucosal barrier integrity.
Fan et al., 2023 [89]; Quagebeur et al., 2023 [90].	*Parabacteroides*	SCFA producer; altered in AN; modulates immunity and metabolism.	Fermentation of complex polysaccharides into SCFAs; activation of anti-inflammatory signaling pathways (e.g., GPR41/43).
Fan et al., 2023 [89].	*Alistipes*	Variable presence in AN; involved in mood and lipid metabolism; potential biomarker.	SCFA production; influence on serotonergic signaling and low-grade inflammation modulation.
Scala et al., 2025 [77].	*Turicibacter*	Altered in AN; involved in immune regulation and serotonin metabolism.	Involvement in immune and metabolic regulation; possible modulation of serotonin synthesis and gut–brain signaling.
Li et al., 2025 [83].	*Klebsiella pneumoniae*	Negatively associated with AN; may exert protective microbial competition.	Production of immunomodulatory metabolites; competes with pathogenic microbes; potential anti-inflammatory role.

Abbreviations: AN, anorexia nervosa; BMI, body mass index; EDs, eating disorders; PPAR-γ, Peroxisome Proliferator-Activated Receptor Gamma; SCFAs, short-chain fatty acids; ↓, decrease, decreased, lower, diminution, worsening, reduced; ↑, increase, augmentation, elevation, improvement, greater.

**Table 2:** Animal and in vitro studies about prebiotics/probiotics supplementation or diet on EDs.

Studies	Study Design	Population	Trial Duration	Type of Intervention	Outcomes
Nicol et al. [206]	Animal study	Male C57Bl6 mice	7 days	1 × 10^10^ CFU mix of *L. salivarius* LS7892 and *L. gasseri* LG6410	Sugar craving in chronic mild stress condition
Trinh et al. [207]	Animal study	Translational activity-based anorexia female Wistar rats	48 days	1 × 10^9^ CFU/mL VSL#3^®^ (*Bifidobacterium breve*, *Bifidobacterium longum*, *Bifidobacterium infantis*, *Streptococcus thermophilus*, *Lactobacillus acidophilus*, *Lactobacillus plantarum*, *Lactobacillus paracasei*, and *Lactobacillus delbrueckii subsp. bulgaricus*)	Gut-associated lymphatic tissue in chronic starvation
Verspohl et al. [208]	Animal study	Translational activity-based anorexia female Wistar rats	35 days	Omega-3 FAs or OMNi-BiOTiC^®^ SR-9 *(Lactobacillus casei W56*, *Lactobacillus acidophilus* W22, *Lactobacillus paracasei* W20, *Bifidobacterium lactis* W51, *Lactobacillus salivarius* W24, *Lactococcus lactis* W19, *Bifidobacterium lactis* W52, *Lactobacillus plantarum* W62, *Bifidobacterium bifidum* W23)	Chronic starvation on glial and neuronal cell populations
Liu et al. [205]	In vitro study	Colonic model	65 days	FOS (1.67 g/daily) or *Saccharomyces boulardii* (5 × 10^8^ CFU)	Dietary restrictions on the intestinal ecosystem

**Table 3:** Human studies about prebiotics/probiotics supplementation or diet on EDs.

Studies	Study Design	Population	Trial Duration	Type of Intervention	Outcomes
Gröbner et al. [209]	Two-center, longitudinal, double-blind, randomized, controlled trial	30 F with AN (13–19 years) compared to 30 in age- and sex-matched placebo group	12 months	Daily OMNi-BiOTiC^®^ SR-9 *(Lactobacillus casei W56*, *Lactobacillus acidophilus* W22, *Lactobacillus paracasei* W20, *Bifidobacterium lactis* W51, *Lactobacillus salivarius* W24, *Lactococcus lactis* W19, *Bifidobacterium lactis* W52, *Lactobacillus plantarum* W62, *Bifidobacterium bifidum* W23)	Gut microbiota composition, weight gain, gastrointestinal complaints, and psychopathology
Ghafouri- Taleghani et al. [210]	Triple-blind, randomized, placebo-controlled clinical trial	25 M/F with food addiction and weight regain after bariatric surgery (18–50 years) compared to 25 F/M in placebo group	12 weeks	1.8 × 10^9^ CFU/capsule multi-strain probiotic (*Lactobacillus acidophilus*, *Bifidobacterium bifidum*, *Bifidobacterium lactis*, *Bifidobacterium longum*, *Lactobacillus reuteri, Lactobacillus rhamnosus*)	Anthropometric measures, biochemical markers, eating behavior, and food addiction
Komorniak et al. [211]	Double-blind, randomized, placebo controlled pilot study	21 M/F adults (≥6 months post-bariatric surgery) with depressive symptoms in the probiotic group and 17 F/M in placebo group	5 weeks	Sanprobi Barrier (*Bifidobacterium bifidum W23*, *Bifidobacterium lactis W51*, *Bifidobacterium lactis W52*, *Lactobacillus acidophilus W37*, *Levilactobacillus brevis W63*, *Lacticaseibacillus casei W56, Ligilactobacillus salivarius W24, Lactococcus lactis W19, and Lactococcus lactis W58)*	Psychometric tests, microbiota composition, and gut barrier markers
Choi et al. [6]	Parallel, double-blind, randomized, placebo-controlled trial	37 M/F with overweight undergoing controlled weight loss (18–55 years) in probiotic group and 30 in placebo group	12 weeks	*Lacticaseibacillus rhamnosus* HA-114 (10 × 10^9^ CFU/capsule)	Eating behaviors, mood-related aspects, and metabolic biomarkers

## Data Availability

Data are contained in the article and cited articles.
